# Chronic Inflammation and Cytokines in the Tumor Microenvironment

**DOI:** 10.1155/2014/149185

**Published:** 2014-05-13

**Authors:** Glauben Landskron, Marjorie De la Fuente, Peti Thuwajit, Chanitra Thuwajit, Marcela A. Hermoso

**Affiliations:** ^1^Disciplinary Program, Institute of Biomedical Sciences, School of Medicine, University of Chile, Independencia 1027, 8380453 Santiago, Chile; ^2^Department of Immunology, School of Medicine, Siriraj Hospital, Mahidol University, 2 Prannok Road, Bangkok Noi, Bangkok 10700, Thailand

## Abstract

Acute inflammation is a response to an alteration induced by a pathogen or a physical or chemical insult, which functions to eliminate the source of the damage and restore homeostasis to the affected tissue. However, chronic inflammation triggers cellular events that can promote malignant transformation of cells and carcinogenesis. Several inflammatory mediators, such as TNF-**α**, IL-6, TGF-**β**, and IL-10, have been shown to participate in both the initiation and progression of cancer. In this review, we explore the role of these cytokines in important events of carcinogenesis, such as their capacity to generate reactive oxygen and nitrogen species, their potential mutagenic effect, and their involvement in mechanisms for epithelial mesenchymal transition, angiogenesis, and metastasis. Finally, we will provide an in-depth analysis of the participation of these cytokines in two types of cancer attributable to chronic inflammatory disease: colitis-associated colorectal cancer and cholangiocarcinoma.

## 1. Introduction

The role of inflammation in the development of cancer was described as early as 1863, by Rudolf Virchow. His observations that inflammatory cells infiltrate tumors led him to hypothesize that cancer arises from inflammatory sites (“lymphoreticular infiltration”) [1, 2]. In the last decades, Virchow's postulation has been supported by abundant evidence that various cancers are triggered by infection and chronic inflammatory disease [3].

Inflammation is a beneficial response activated to restore tissue injury and pathogenic agents. However, if inflammation is unregulated, it can become chronic, inducing malignant cell transformation in the surrounding tissue. The inflammatory response shares various molecular targets and signaling pathways with the carcinogenic process, such as apoptosis, increased proliferation rate, and angiogenesis. Furthermore, the use of nonsteroidal anti-inflammatory drugs (NSAIDs) has been shown to decrease incidence and mortality of several cancers [4].

In relation to chronic inflammatory-associated neoplasias, this review article explores the involvement of cytokines in chronic inflammation and carcinogenesis, focusing on inflammatory bowel disease-associated cancer and cholangiocarcinoma (CCA) induced by chronic inflammation of biliary ducts, that is, primary sclerosing cholangitis (PSC) and liver fluke associated-CCA. Both cancers are examples of a localized, long-term inflammatory process increasing the risk of cancer.

## 2. Chronic Inflammation as an Inducer of Tumors

The immune response comprises a series of events triggered in response to recognition of pathogens or tissue damage, involving cells and soluble mediators, such as cytokines of the innate and adaptive immune system. The main purpose of this inflammatory response is to remove the foreign agent disturbing tissue homeostasis [5]. In the normal physiological context, after tissue repair or pathogen elimination, the inflammation is resolved and the homeostatic state recovered [6].

It is now widely accepted that inadequately resolved chronic inflammation may increase the risk of cancer. Several pathologies illustrate this link, such as endometriosis, chronic prostatitis, and chronic gastritis due to* Helicobacter pylori *(*H. pylori*), inflammatory bowel diseases (IBD), and primary sclerosing cholangitis (PSC) (Table 1). Inflammation can increase the risk of cancer by providing bioactive molecules from cells infiltrating the tumor microenvironment, including cytokines; growth factors; chemokines that maintain a sustained proliferative rate; cell survival signals to avoid apoptosis; proangiogenic factors; and extracellular matrix-modifying enzymes such as metalloproteinases that promote epithelial-mesenchymal transition (EMT) and facilitate other carcinogenesis programs, such as genome instability, reprogramming of energy metabolism, and immune evasion [7]. Here, we focus on key cytokines involved in tumor induction and their role in EMT, angiogenesis, invasion, and metastasis.

## 3. Cytokines Involved in Tumor Development 

Cytokines are low-molecular-weight proteins that mediate cell-to-cell communication. Immune and stromal cells, such as fibroblasts and endothelial cells, synthesize them and they regulate proliferation, cell survival, differentiation, immune cell activation, cell migration, and death. Depending on the tumor microenvironment, cytokines can modulate an antitumoral response, but during chronic inflammation, they can also induce cell transformation and malignancy, conditional on the balance of pro- and anti-inflammatory cytokines, their relative concentrations, cytokine receptor expression content, and the activation state of surrounding cells [50].

### 3.1. Tumor Necrosis Factor (TNF-*α*)

As noted, unresolved inflammation can lead to malignancy. Tumor necrosis factor (TNF-*α*) is one inflammatory mediator that has been implicated in carcinogenesis, due to its participation in chronic inflammatory diseases [51]. Moore et al. provided evidence that TNF-*α*-deficient mice are resistant to tetradecanoyl-phorbol-13-acetate- (TPA-) induced skin carcinogenesis. TNF-*α* effect seems to be more significant in the early stages of carcinogenesis, including angiogenesis and invasion, versus progression of carcinogenesis [52, 53]. While TNF-*α* is a prototypical proinflammatory cytokine, evidence suggests a double-edged role in carcinogenesis. This cytokine is recognized by two receptors: TNF-*α* receptor-1 (TNF-*α*R-1), ubiquitously expressed, and TNF-*α*R-2, expressed mainly in immune cells [54]. Trimerization occurs upon TNF-*α* binding to TNF-*α*-Rs, leading to activation of at least four signaling pathways: a proapoptotic pathway induced by caspase-8 interaction with Fas-associated death domain (FADD); an antiapoptotic platform activated by cellular inhibitor of apoptosis protein-1 (cIAP-1) and interacting with TNF-*α*R-associated factor 2 (TRAF2); a TRAF2- and JNK-mediated AP-1 signaling pathway; and a receptor interacting protein- (RIP-) induced NF-*κ*B [54].

There is controversy, however, regarding the role of TNF-*α* in cancer; high concentrations of this cytokine can induce an antitumoral response in a murine model of sarcoma [55]. Furthermore, William B. Coley, a pioneer surgeon in the field, discovered that there was a reliable treatment response for systemic bacterial filtrate injection in sarcoma patients [55, 56]. However, severe toxic side effects have been associated with systemically administered TNF-*α*, such as hypotension and organ failure [57]. Local administration has been shown to be safer and effective, as demonstrated by clinical trials evaluating a TNF-*α*-expressing adenovirus (TNFerade) gene therapy combined with chemotherapy [58, 59]. Moreover, TNF-*α*-conjugate targeting peptides or single-chain antibody fragments have also shown variable effects, depending on the patient [60].

In contrast, low, sustained TNF-*α* production levels can induce a tumor phenotype [61]. A TNF-*α* tumor promotion mechanism is based on reactive oxygen species (ROS) and reactive nitrogen species (RNS) generation, which can induce DNA damage, hence facilitating tumorigenesis [62, 63]. TNF-*α*-mediated inflammation has been linked to cancer; for instance, increased TNF-*α* levels in preneoplastic lesions have been detected in* H. pylori*-positive gastric lesions, through* H. pylori*-secreted TNF-*α*-inducing protein (Tip*α*) [64, 65].

A study by Kwong et al. explored TNF-*α*-associated tumorigenesis using an organoid of normal human ovarian epithelial cells exposed to a prolonged TNF-*α* dose. The model demonstrated generation of a precancerous-like phenotype with structural and functional changes, such as tissue disorganization, epithelial polarity loss, cell invasion, and overexpression of cancer markers [66].

According to these findings, the pro- or antitumoral TNF-*α* response within the tumor microenvironment depends not only on local concentration but also on its expression site in the tumor. Patients with elevated levels of TNF-*α* in tumor islets from non-small cell lung cancer, mainly restricted to macrophages and mast cells, showed the highest survival rates, while patients with increased stromal TNF-*α* content showed lower survival rates [67].

There is also evidence that prolonged TNF-*α* exposure can enhance the proportion of cancer stem cell phenotypes in oral squamous cell carcinoma, increasing their tumor-forming sphere ability, stem cell-transcription factor expression, and tumorigenicity [68].

### 3.2. Interleukin 6 (IL-6)

Another proinflammatory cytokine with a typical protumorigenic effect is IL-6. Elevated serum IL-6 levels have been detected in patients with systemic cancers as compared to healthy controls or patients with benign diseases. IL-6 has been proposed as a malignancy predictor, with sensitivity and specificity of about 60–70% and 58–90%, respectively [69]. However, there are limited studies available that might be used to define cut-off values for IL-6 as a diagnostic tool.

IL-6 plays a key role in promoting proliferation and inhibition of apoptosis, by binding to its receptor (IL-6R*α*) and coreceptor gp130 (glycoprotein 130), thus activating the JAK/STAT signaling pathway of the Janus kinases (JAK) and signal transducers and activators of transcription (STATs) STAT1 and STAT3 [70]. STATs belong to a family of transcription factors closely associated with the tumorigenic processes. Several studies have highlighted the effect of the IL-6/JAK/STAT signaling pathway on cancer initiation and progression. IL-6 can induce tumorigenesis by hypermethylation of tumor suppressor genes as well as by hypomethylation of retrotransposon long interspersed nuclear element-1 (LINE-1) in oral squamous cell cancer lines* in vitro* [71], a frequent event in various human cancers. Furthermore, IL-6 has been shown to be produced primarily by stromal fibroblasts in a gastric cancer mouse model; however, the deficient mouse model exhibits reduced tumorigenesis when exposed to the carcinogen N-methyl-N-nitrosourea [72].

IL-6 has a role in multiple myeloma development, as demonstrated by its ability to induce apoptosis by blocking the IL-6R/STAT3 pathway* in vitro* [73] and the resistance of IL-6^−/−^ mice to plasmacytoma induction [74].

Like TNF-*α*, IL-6 facilitates tumor development by promoting conversion of noncancer cells into tumor stem cells. In particular, IL-6 secretion by noncancer stem cells in low-attachment culture conditions upregulates Oct4 gene expression by activating the IL-6R/JAK/STAT3 signaling pathway [75].

These findings have led researchers to propose IL-6 as a therapeutic target in cancer. Several phase I/II clinical trials are currently evaluating antibodies against IL-6 or IL-6R as therapeutic alternatives. Siltuximab (CNTO 328), a monoclonal antibody against IL-6, has shown promising results for non-small cell lung cancer, ovarian cancer, prostate cancer, and multiple myeloma, among others [76–80].

In this context, as inflammatory cytokines are partially responsible for tumor induction, an increase in anti-inflammatory cytokines should limit the risk of cancer and reduce activation of signaling pathways. Nonetheless, evidence suggests that anti-inflammatory cytokines, such as TGF-*β* and IL-10, show more complex effects on tumor development.

### 3.3. Transforming Growth Factor *β* (TGF-*β*)

TGF-*β* is a powerful pleiotropic cytokine, with immune-suppressing and anti-inflammatory properties. Under physiological conditions, TGF-*β* has a well-documented role in embryogenesis, cell proliferation, differentiation, apoptosis, adhesion, and invasion [81]. Three isoforms have been identified: TGF-*β*1, TGF-*β*2, and TGF-*β*3. TGF-*β*s binds to the cognate type II receptor (TGF-*β* RII), inducing type I TGF-*β* receptor (TGF-*β* RI) phosphorylation and leading to the formation of a heterotetrameric complex that activates SMAD-dependent transcription [82]. SMAD transcription factors are structurally formed by a serine and threonine-rich linker region that connects two MAD (mothers against dpp) homology regions. Differential phosphorylation of these amino acid residues contributes to various cellular functions, including cytostatic effects, cell growth, invasion, extracellular matrix synthesis, cell cycle arrest, and migration [83]. Therefore, differential phosphorylation of SMAD2 and SMAD3 by TGF-*β* receptor activation promotes their translocation into the nucleus, where they form a complex with SMAD4, further bind to DNA, associate with other transcription factors, and induce gene expression [82].

The role of TGF-*β* in cancer is complex and paradoxical, varying by cell type and stage of tumorigenesis. In early stages, TGF-*β* acts as a tumor suppressor, inhibiting cell cycle progression and promoting apoptosis. Later, TGF-*β* enhances invasion and metastasis by inducing epithelial-mesenchymal transition (EMT) [84]. In cancer induction, TGF-*β* exerts a tumor suppressor effect through cyclin-dependent kinase inhibitor (CKI) p21 upregulation and c-Myc downregulation [85]. Using a conditional TGF-*β* RII knock-out mice model, Guasch et al. found that highly proliferative epithelia (such as rectal and genital) developed spontaneous squamous cell carcinomas and furthermore showed accelerated carcinoma progression, Ras mutations, and apoptosis reduction [86], suggesting that a deficient TGF-*β* pathway contributes to tumorigenesis.

There is consistent evidence demonstrating that TGF-*β* signaling changes are involved in human cancer. Increased TGF-*β*1 mRNA and protein have been observed in gastric carcinoma, non-small cell lung cancer, and colorectal and prostate cancer [87], and TGF-*β* receptor deletion or mutations have been associated with colorectal, prostate, breast, and bladder cancer, correlating with a more invasive and advanced carcinoma, higher degree of invasion, and worse prognosis [88].

In the tumor microenvironment, common sources of TGF-*β* are cancer and stromal cells, including immune cells and fibroblasts [82]. Bone matrix is also an abundant source of TGF-*β* and a common site for metastasis in many cancers, correlating with the tumor-promoting and invasive effects of this cytokine [89].

Specific therapy targeting this cytokine in advanced cancer patients has shown promising results in preclinical and clinical studies, using TGF-*β* inhibitors, specifically ligand traps, antisense oligonucleotides, receptor kinase inhibitors, and peptide aptamers. Nevertheless, serious side effects of systemic TGF-*β* inhibitors administration have been reported, indicating that further clinical trials are required to evaluate localized, safe, dose-effective therapies [89].

### 3.4. Interleukin 10 (IL-10)

Interleukin 10 (IL-10) is known to be a potent anti-inflammatory cytokine. Almost all immune cells, including T cells, B cells, monocytes, macrophages, mast cells, granulocytes, dendritic cells, and keratinocytes, produce IL-10 [90]. Tumor cells can also secrete IL-10, as can tumor-infiltrating macrophages [91, 92].

When IL-10 binds to its receptor, Jak1 and Tyk2 tyrosine kinases phosphorylate an IL-10R intracellular domain, allowing it to interact with STAT1, STAT3, and STAT5, favoring STAT translocation into the nucleus and induction of target gene expression [93].

Several studies have indicated that IL-10 has both pro- and antitumoral effects. IL-10 inhibits NF-*κ*B signaling; therefore, it can downregulate proinflammatory cytokine expression [94] and act as an antitumoral cytokine. Consistent with this finding, Berg et al. demonstrated that IL-10-deficient murine models are prone to bacteria-induced carcinogenesis [95], whereas the adoptive transfer of IL-10-expressing CD4^+^CD25^+^ T cells into Rag2^−/−^ (lymphocyte-deficient) mice inhibits colorectal inflammation and carcinomas [96, 97]. Moreover, IL-10 can exert antitumoral activity in gliomas, melanomas, and breast and ovarian carcinomas [98], through a mechanism involving MHC-I downregulation, thus inducing NK-mediated tumor cell lysis [99].

Due to its immunosuppressive effect on dendritic cells and macrophages, IL-10 can dampen antigen presentation, cell maturation, and differentiation, allowing tumor cells to evade immune surveillance mechanisms [100].

In addition and as previously described for IL-6, STAT3 can also be activated by IL-10, although the cytokines' contradictory responses are determined by receptor and time frame of STAT activation. In particular, IL-6 leads to a transient, rapidly declining STAT3 phosphorylation and nuclear localization, whereas IL-10 induces a sustained STAT3 phosphorylation [101]. Through STAT3 activation, IL-10 can also have a protumorigenic effect, mediated by an autocrine-paracrine loop [102] involving Bcl-2 upregulation and apoptosis resistance activation [103, 104]. Likewise, elevated IL-10 levels are associated with poor prognosis in diffuse B cell lymphoma [105] and expression by tumor cells, and tumor-associated macrophages promote Burkitt's lymphoma through the increased production of a TNF-*α* family member, BAFF, a tumor growth/survival molecule [106].

## 4. Inflammatory Response and Malignancy

### 4.1. Inflammation-Induced Reactive Oxygen Species (ROS) and Reactive Nitrogen Species (RNS) in the Carcinogenic Process

In an inflammatory response, epithelial and immune cell activation trigger ROS and RNS generation through induction of NADPH oxidase and nitric oxide synthase (NOS), respectively. NADPH oxidase is a protein complex composed of several membrane-associated subunits that catalyze the superoxide anion (O_2_^−•^), leading to superoxide dismutase- (SOD-) mediated hydrogen peroxide (H_2_O_2_) production. NADPH oxidase is expressed in phagocytic and nonphagocytic cells, and cytochrome subunit isoforms are present in different cell types (NOX2 in phagocytic cells, such as macrophages and neutrophils) (NOX1, 3–5, and DUOX1, 2 in nonphagocytic cells) [107]. On the other hand, NOS generates nitric oxide (NO) from L-arginine, which can be converted into RNS such as nitrogen dioxide (NO_2_^•^), peroxynitrite (ONOO^−^), and dinitrogen trioxide (N_2_O_3_). Different NOS isoforms are produced depending on cell type: inducible NOS (iNOS) in phagocytic cells and constitutive in endothelial and neuronal (eNOS and nNOS) cells [108]. ROS and RNS have a potent antimicrobial role in phagocytic cells and also act as a second messenger in signaling transduction [109, 110].

Phagocytic cell activation can directly induce reactive oxygen and nitrogen species (collectively called RONS), activating NOX2, NADPH oxidase, and iNOS [109]. Furthermore, TNF-*α*, IL-6, and TGF-*β* trigger RONS generation in nonphagocytic cells [111–113].

Increased expression of NADPH oxidase and NOS and their products RONS has been identified in various cancers, suggesting that free radicals have a role in genesis and malignant progression [63]. In various chronic inflammatory diseases, such as* H. pylori*-associated gastritis and inflammatory bowel diseases (IBD), high RONS levels have been observed, suggesting a role in cancer risk [114–116].

**Figure 1:**
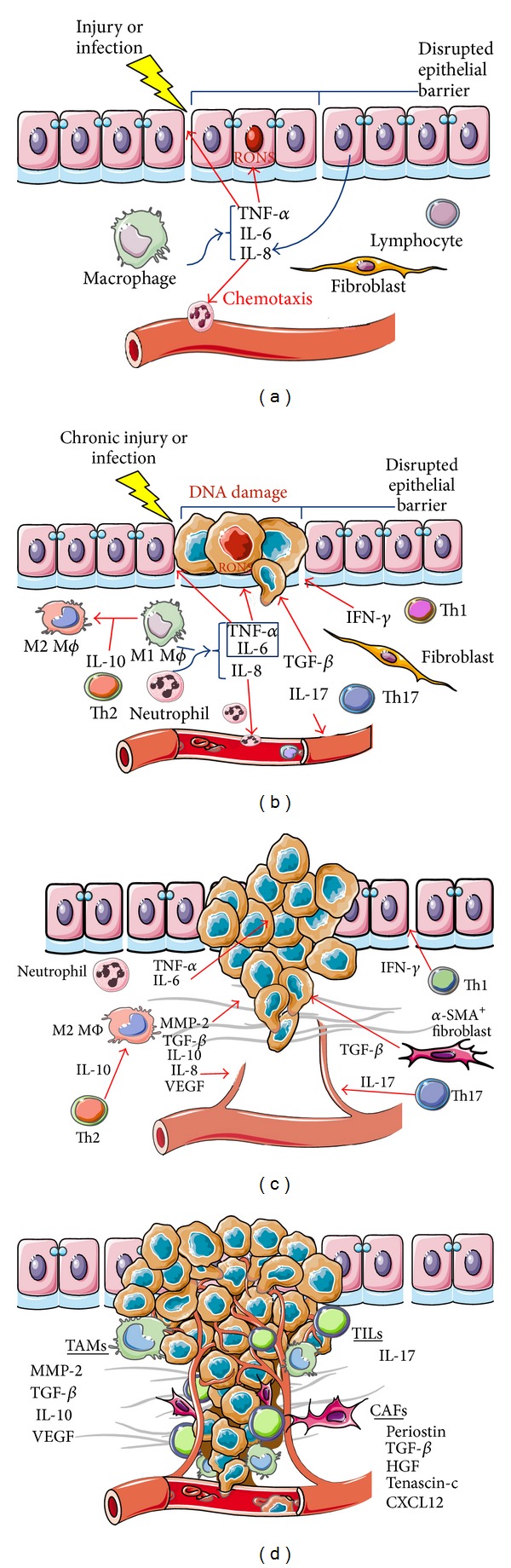
Schematic illustration of the role of cytokines in carcinogenesis. (a) During tissue injury or infection, an immune response activates the expression of proinflammatory mediators, such as TNF-*α*, IL-6, and IL-8 from macrophages and neutrophils. These cytokines can disrupt the epithelial barrier, induce RONS, and promote the infiltration of other inflammatory cells. (b) In chronic inflammation, proinflammatory cytokines such as TNF-*α* can induce DNA damage through RONS, which leads to tumor initiation. TGF-*β* can promote malignant transformation through EMT activation. Cytokines derived from CD4^+^lymphocytes, such as IFN-*γ*, IL-10, and IL-17, can participate in epithelial barrier disruption, M2 phenotypic transitions of macrophages, and angiogenesis, respectively. (c) Tumor growth and invasion are also favored by proinflammatory cytokines that stimulate cell proliferation, reduce apoptosis, and enhance EMT and angiogenesis; the latter is facilitated by VEGF and IL-8. Anti-inflammatory cytokines, such as IL-10 and TGF-*β*, contribute to tumor immune evasion. (d) Tumor-associated macrophages (TAM), tumor-infiltrating lymphocytes (TIL), and cancer-associated fibroblasts (CAF) secrete several factors that contribute to tumor growth and metastasis, while maintaining the immunosuppressive milieu.

Different mechanisms have been proposed to clarify RONS participation in cancer development. RONS induce cell oxidative stress and damage of lipids, proteins, and DNA, as well as production of 8-oxo-7, 8-dihydro-2′-deoxyguanosine (8-oxodG), which is actually used as a DNA damage marker. Furthermore, 8-oxodG can pair with adenine, leading to transversion of G:C to T:A (G→T transversion). Similarly, ONOO^−^ can modify deoxyguanosine to 8-nitrodeoxyguanosine, which can spontaneously generate an apurinic site, favoring G→T transversion [19]. Identification of these DNA damage markers in chronic inflammatory processes, such as* H. pylori*-associated gastritis, hepatitis, and ulcerative colitis, emphasizes the relevance of RONS in pathologies with an increased risk of cancer (Figures 1(a) and 1(b)) [19, 117, 118]. Moreover, 8-oxodG and 8-nitrodeoxyguanine immune-reactivity is increased in the liver of hepatitis C virus-derived chronic hepatitis patients [118].

Jaiswal et al. found increased iNOS, 3-nitrotyrosine, and 8-oxodG in the livers of primary sclerosis cholangitis (PSC) patients [119]. Furthermore, RNS interfere with DNA repair, as shown in cells overexpressing iNOS that are unable to repair modified 8-oxodG [119]. Deficient DNA-repair protein activity has been linked to enzyme S-nitrosylation, attributable to increased RNS [120].

RONS are generated by cellular stress and macromolecule modification, although they are also involved in the regulation of signaling pathways, such as survival and cell proliferation through Akt, Erk1/2, and hypoxia-inducible factor-1 (HIF-1) activation [121, 122].

There is strong evidence linking carcinogenesis to inflammatory response and RONS, and therapeutic strategies for cancer prevention using free radicals and proinflammatory signaling inhibitors have been evaluated in animal models [123–125].

### 4.2. Inflammation-Associated Tumor Growth

Nowadays, it is accepted that chronic inflammation is important in generating malignancy through the exposure of proinflammatory cytokines and sustained activation of signaling pathways such as NF-*κ*B and STAT3. Following cell transformation to a malignant state, these cytokines are also involved in tumor growth, by stimulating the proliferation of tumor cells and by evading immunosurveillance (Figures 1(b) and 1(c)).

Several cytokines have growth factor activity; a relevant cytokine is TNF-*α*. In a study by Zhu et al., they showed that the silencing of TNF-*α* in a gallbladder cell line decreases cell proliferation and invasion by an autocrine effect, affecting the activation of TNF-*α*/NF-*κ*B/AKT/Bcl-2 pathway in these cells [126]. This is consistent with data previously observed by Luo et al. who revealed that NF-*κ*B signaling is required to promote tumor cell proliferation in response to an inflammatory stimulus, and by inhibiting this transcription factor, an antitumor signal led by TNF-*α*/TRAIL is triggered [20]. However, in a mouse model of ovarian cancer, TNF-*α* can also stimulate the secretion of other cytokines like IL-17 by CD4^+^ T cells and promote tumor growth indirectly [127].

The protumorigenic role of IL-17 has also been implicated in other types of cancer. In mice with carcinogen-induced skin tumors, those deficient in IL-17 receptor showed a lower tumor incidence and a diminished tumor size [128].

IL-6 is another typical proinflammatory cytokine with tumor growth effect, mainly by activating JAK tyrosine kinases and the transcription factor STAT3, as seen in lung, kidney, and breast cancer in which a high expression of STAT3 has been identified [70]. Also, in cell lines of malignant fibrous histiocytoma, a high secretion of IL-6 and constitutive activation of STAT3 were reported, reflecting an increase of tumor cell proliferation [129].

In cancer, other molecules that may influence tumor growth by regulating the IL-6/STAT3 signaling pathway have been reported. Inflammatory mediators like Hmgb1, IL-23, and IL17 can promote tumor growth by activating IL-6/STAT3 pathway in a mouse model of melanoma [130]. In cholangiocarcinoma, a high expression of the tumor suppressor gene regulator, gankyrin, favors tumor proliferation, invasion, and metastasis through activation of IL-6/STAT3 signaling pathway [131]. Furthermore, embelin, a derivative from* Embelia ribes*, is known to inhibit XIAP (X-linked inhibitor of apoptosis protein) and is able to impair tumor proliferation by interfering in IL-6/STAT3 signaling [132].

Finally, the anti-inflammatory cytokine IL-10 may also contribute to tumor growth. In a mouse model of melanoma, tumors overexpressing IL-10 present a higher tumor growth mediated by an increase in tumor cell proliferation, angiogenesis, and immune evasion [133].

### 4.3. Inflammation-Associated Epithelial Mesenchymal Transition

The epithelial mesenchymal transition (EMT) is an important process of cellular reprogramming during embryogenesis and pathological events such as inflammation, wound healing, and cancer [134, 135]. During EMT, epithelial cells exhibit morphological changes, acquiring fibroblast characteristics. In this process, structures involved in epithelial cell-cell interaction, such as tight junctions, adherens junctions, desmosomes, and gap junctions, are lost, and the cells undergo actin cytoskeleton reorganization and changes in the expression profile of proteins allowing for cell-cell contact, such as E-cadherin. Furthermore, expression of fibroblast markers, including fibronectin, *α*-smooth muscle actin (*α*-SMA), and matrix metalloproteinases, is favored during EMT. Cellular reprogramming is orchestrated by a variety of transcription factors, such as Snail, ZEB, and the helix-loop-helix (HLH) family [136, 137]. The mesenchymal phenotype provides increased motility that is associated with invasiveness and metastasis of tumor cells [138, 139].

One inflammatory mediator relevant in EMT is TGF-*β*, as demonstrated by its role in embryogenesis, fibrosis, and tumor development in various EMT models [137, 140–142]. SMAD2, SMAD3, and SMAD4 mediate EMT modulation via TGF-*β* signaling [137], as shown by EMT inhibition in SMAD3-deficient mice and by SMAD2-, SMAD3-, or SMAD4-dominant negative constructs* in vitro* [143, 144]. Extensive evidence supports the notion that EMT can be induced by proinflammatory cytokines. TNF-*α* and IL-6 may synergistically nudge the TGF-*β* signaling pathway towards EMT progression (Figures 1(b) and 1(c)) [21, 145]. Both cytokines promote NF-*κ*B activation, which regulates the expression of transcription factors involved in EMT, orchestrating the effects of Snail1, Snail2, Twist, ZEB1, and ZEB2 [146, 147]. Moreover, IL-6 induces cell invasiveness in EMT, through increased vimentin and downregulated E-cadherin expression, both mediated by the JAK/STAT3/Snail signaling pathway, as shown in head and neck cancer [148].

Finally, ROS production can promote EMT [149]; therefore, exposing kidney epithelial cells to ROS induces TGF-*β* expression, the SMAD signaling pathway, and EMT, whereas antioxidants inhibit these processes [150].

### 4.4. Inflammation-Associated Angiogenesis

Angiogenesis comprises the processes leading to the generation of new blood vessels from an existing vascular network. Angiogenesis in cancer development is important because the new blood vessel network penetrates and supplies nutrients and oxygen to tumor cells. Several angiogenic factors secreted by tumor cells have been identified, in particular vascular endothelial growth factor (VEGF) that is expressed in response to cytokines and growth factors, as shown in Figures 1(c) and 1(d) [151]. Moreover, characterization of tumor-associated macrophages (TAM) obtained from metastatic lymph nodes (MLN) in an animal model of melanoma has shown that MLN are constituted predominantly by TIE2^+^/CD31^+^ infiltrating macrophages. This subpopulation significantly overexpresses VEGF and is directly related to angiogenesis [152].

Fajardo et al. showed that TNF-*α* might have a double-edged role in angiogenesis, depending on the dose used. High TNF-*α* doses inhibited angiogenesis in mice subcutaneously implanted with an angiogenesis disc-system, an experimental strategy used to induce new blood vessels, while low doses promoted vascularization of the area [153]. The antiangiogenic effect of TNF-*α* is related to downregulation of *ανβ*3 and the angiotensin signaling pathway [154], while proangiogenic responses have been associated with increased VEGF, VEGFR, IL-8, and FGF expression [155].

On the other hand, low TNF-*α* levels increase tumor growth, induce angiogenesis of diverse tumors in mice, and induce a subpopulation of tumor-associated myeloid cells coexpressing endothelial and myeloid markers with proangiogenic/provasculogenic properties [156].

The tumor source of TNF-*α* can be derived from myeloid or tumor cells and through an autocrine activation can stimulate tumor growth and angiogenesis [157]. Likewise, tumors derived from TNF-*α* knockdown cells have a well-circumscribed phenotype, with low vascularization and less invasiveness [157].

Another relevant angiogenic factor is IL-6; high levels correlate with VEGF content in colorectal and gastric cancer [158, 159]. Moreover, IL-6 induces VEGF expression in a dose-dependent manner in gastric cancer cell lines [160]. Similarly, IL-6 promotes angiogenesis by activating the STAT3 pathway in cervical cancer [161]. Together, IL-6 secretion and the subsequent STAT3 phosphorylation are involved in the upregulation of angiogenic mediators, such as VEGF, HIF1*α*, the VEGFR2 coreceptor, and neuropilin 2 (NRP2) [162, 163]. In xenograft models of ovarian cancer, reduced tumor neovascularization, TAM infiltration, and chemokine production were demonstrated after a challenge with siltuximab, a high-affinity anti-IL-6 antibody [77].

A proangiogenic effect has also been attributed to TGF-*β* [88]. High TGF-*β* levels in tumors correlate with angiogenesis in prostate cancer [164]. In addition, TGF-*β* levels correlate with VEGF expression in gastric carcinoma [165]. These data are consistent with the defective vasculogenesis shown in TGF-*β*1 knockdown mice [166].

On the other hand, anti-inflammatory IL-10 has been suggested to have an antiangiogenic role in several cancer models [167, 168]. Overexpression of mIL-10 in the KOC-2S tumor cell line had little effect on the VEGF-hyposecretory phenotype, suggesting that mIL-10-mediated inhibition of angiogenesis is mediated by VEGF [169].

### 4.5. Inflammation-Associated Metastasis

Metastasis is a process characterized by neoplastic cell spread to another organ of different origin. During metastasis, the cells invade blood and lymphatic vessels and circulate through the bloodstream, with subsequent retention in another organ, generating a new tumor focus.

The metastatic cascade is modulated by the action of several cytokines released by surrounding cells such as tumor associated macrophages, infiltrating lymphocytes, and cancer associated fibroblasts, promoting tumor cell evasion and dissemination; this process is depicted in Figure 1(d). The influence of TNF-*α* has been investigated in various experimental animal models. Administration of this cytokine leads to a significant increase of the number of lung metastases [170, 171]. Kim et al. proposed that tumor cells activate myeloid cells to generate a microenvironment favorable for metastasis. In Lewis lung carcinoma (LLC) cell conditioned-medium, high levels of IL-6 and TNF-*α* were induced in bone marrow-derived macrophages [172]. TNF-*α* ^−/−^ but not IL-6^−/−^ mice injected with LLC cells showed improved survival and reduced lung tumor multiplicity, suggesting a critical role of TNF-*α* in LLC metastasis [172]. In accordance with these data, studies show that the use of anti-TNF-*α* antibodies aids in decreasing metastasis [4, 173]. IL-6, in turn, is upregulated in various tumors and has been implicated in the capacity of cancer cells to metastasize to bone [148, 174, 175].

In contrast, IL-10 displays an antitumoral function. Restitution of IL-10 in the A375P human melanoma cell line, which does not produce endogenous IL-10, using a vector containing murine IL-10 cDNA, reverted tumor growth and lung metastases. This evidence suggests that IL-10 production by tumor cells inhibits metastasis [167].

There is a strong relationship between EMT and metastasis, suggesting that, in the early stages of the metastatic cascade, EMT enables migration and intravasation of tumor cells [176]. For this reason, inflammatory mediators involved in EMT, in particular TGF-*β*, might play an important role in promoting metastasis [138].

## 5. Colorectal Cancer and Inflammatory Bowel Disease

Colorectal cancer is the third-most frequent cancer worldwide, with a higher incidence in developed countries [177]. A mortality rate of about 9% has been reported for both men and women, with 5-year survival between 74% and 59% for early stages (stages I to IIC) and 6% for stage IV [178].

Today it is widely accepted that IBD patients have a higher risk of CRC especially ulcerative colitis (UC) and to a much lesser extent Crohn's disease (CD). In a population-based study in the United States, standardized incidence ratios (SIR) of 2.4 (95% IC 0.6–6.0) in extensive UC or pancolitis and 1.9 in CD (95% IC 0.7–4.1) were reported [8]. The prevalence of CRC in UC patients in the Asia-Pacific region ranges from 0.3 to 1.8% [179]. In a Japanese study, poorer survival was observed in patients with ulcerative colitis-associated colorectal cancer as compared to sporadic colorectal cancer patients in advanced stages [180].

Risk factors involved in this process include a greater extent of compromised tissue and sustained disease duration with an onset of more than 7 years, with risk increasing 0.5–1.0% per year [181]. Another risk factor is concomitant primary sclerosing cholangitis (PSC) and UC, with an OR 4.79: 95% CI (3.58, 6.41) [182].

As noted previously, several types of cancer are associated with chronic infections (Table 1). The IBD are multifactorial pathologies involving changes in the microbiota, possibly attributable to pathogens such as* Mycobacterium avium paratuberculosis* and adherent-invasive* Escherichia coli* [183]. These pathogens can induce an inflammatory response [184–186], which may be associated with higher risk of carcinogenesis; however, more studies demonstrating the chronicity of these infections in IBD patients and their potential role in carcinogenesis are needed.

Various murine models of colitis-associated cancer (CAC) [187] have elucidated much of the carcinogenic process, such as a genetic model of IL-10-deficient mice that develop spontaneous colitis and colonic neoplasms [44] and a DSS-induced colitis and carcinoma model. DSS is a mucosal irritant that induces damage similar to that seen in UC patients, and, through a dose-repeated regimen, DSS-exposed mice develop tumors [188, 189]. An additional chemically induced murine model involves an azoxymethane (AOM) stimulus combined with repeated DSS doses. AOM is a mutagenic agent favoring mutation of the *β*-catenin protooncogene, inducing localization to the nucleus and increasing iNOS and cyclooxygenase (COX-2) expression [190, 191]. Through the animal models, we have learned that inflammatory cytokines, chemokines, and growth factors play crucial roles in CAC development. However, these models have limitations, as they do not always represent the complexity of the mechanisms involved in CRC-IBD patients [187].

In IBD, many inflammatory cytokines are involved in carcinogenesis, such as TNF-*α* and IL-6 (Table 2). In untreated UC patients, mucosal TNF-*α* levels correlate with the degree of swelling [192]. Furthermore, high IL-6 levels have been observed in intestinal biopsies from active IBD patients [193], and murine models have demonstrated a crucial role for these two relevant proinflammatory cytokines in the initiation and progression of CAC [33, 194].

As noted above, proinflammatory cytokines can induce the generation of RONS, a process that has been observed in IBD patients [115], increasing the risk of carcinogenesis [195] by promoting oxidative stress-mediated DNA damage [19]. High ROS levels induced by chronic inflammation have been associated with early p53 mutations in CAC, distinguishing it from sporadic colorectal cancer, in which these mutations have been identified in later stages of malignancy [196]. Thus, the mutagenic potential of RONS, together with early mutations of the p53 tumor suppressor gene, has the potential to increase the cumulative risk associated with genetic alterations predisposing to carcinogenesis in UC patients.

There is abundant evidence for the role of EMT in CAC progression and the participation of TGF-*β* in EMT [38]. Patients with IBD or CRC show elevated TGF-*β* levels [197, 198]. In an IL-10-deficient CAC murine model, incidence of colorectal carcinoma was 65% at the age of 10–31 weeks, and plasma TGF-*β* levels were higher than in their wild-type littermates [44]. Through* in vitro* assays, a well-differentiated colon carcinoma cell line LIM1863 was shown to undergo EMT conversion with a migratory monolayer phenotype in response to TGF-*β*. Moreover, TNF-*α* stimulates IL-8 expression, which in turn accelerates TGF-*β*-induced EMT [21]. Therefore, a proinflammatory stimulus favors the invasive properties of CAC, potentiating EMT.

As previously detailed, angiogenesis is a relevant process in carcinogenesis. Mucosal tissue from IBD patients shows higher microvessel density, a process associated with increased expression of VEGF-induced inflammation [22, 199]. Concomitantly, the CAC mouse model replicated the higher VEGF activity, and blockade of VEGFR2 suppressed tumor development, angiogenesis, and cell proliferation [200].

Furthermore, in an experimental murine cancer metastasis model in which tumor growth was stimulated by bacterial lipopolysaccharide (LPS) injection, TNF-*α*-induced NF-*κ*B signaling in tumor cells was essential for the generation of metastasis. Moreover, NF-*κ*B blockade resulted in reversion of LPS-induced tumor growth [20]. Taken together, these effects of NF-*κ*B signaling indicate that it is a decisive pathway for driving metastasis.

A recently described molecule involved in metastasis is periostin, an extracellular matrix protein secreted in response to mechanical stress and tissue repair by pericryptal and cancer associated fibroblasts (CAFs). Periostin is expressed in invasive front of colon carcinoma, suggesting its participation in tumor growth [201]. Periostin expression dramatically enhances metastatic growth of colon cancer by both preventing stress-induced apoptosis in cancer cells and augmenting endothelial cell survival to promote angiogenesis [202].

The inflammatory process associated with carcinogenesis in CAC is not limited to the above-mentioned cytokines. Other inflammatory mediators are also involved, such as the proinflammatory cytokine IL-17, which was found to be elevated in the mucosa and serum of active IBD patients [203]. Furthermore, IL-17 is overexpressed in tumors from CAC patients and is associated with angiogenesis and poor prognosis markers [46]. The protumorigenic role of IL-17 has also been observed in a IL-17-deficient mouse model of CAC induced with AOM and DSS, where minor tumor formation and a decrease in proinflammatory markers were found for the IL-17-deficient mice as compared to wild-type mice [204].

Another proinflammatory cytokine with a role in CAC is IL-21, which is elevated in the mucosa of IBD patients and in the CAC mouse model [49]. Furthermore, blockade of the IL-21 signaling pathway reduces tumor development and mucosal microenvironment inflammation [49].

Interferon-*γ* (IFN-*γ*) is a proinflammatory cytokine with pleiotropic functions [205]. Increased numbers of IFN-*γ* positive cells have been observed in IBD patients, especially Crohn's disease [27], possibly contributing to a chronic inflammatory setting. Moreover, IFN-*γ*-deficient mice did not develop DSS-induced colitis [28]. In early IBD pathogenesis, IFN-*γ* plays an important role in increasing paracellular permeability in T84 epithelial cells by inducing endocytosis of tight-junction (TJ) proteins occludin, JAM-A, and claudin-1 [29]. In an IL-10-deficient model, enterocolitis and tumor formation were dependent on the participation of IFN-*γ*, as blockage with a neutralizing antibody prevented colitis and cancer in young mice (less than 3 weeks old). However, this effect was not seen in mice older than 3 months, emphasizing the role of IFN-*γ* as an early inducer of inflammation [95].

In an AOM/TNBS-CAC murine model, Osawa et al. showed that IFN-*γ* ^−/−^ mice developed higher numbers of tumors than wild-type or IL-4^−/−^ mice. This points to the antitumor immune response of IFN-*γ* [30]. In patients with UC-associated cancer and a group of UC patients with chronic severe inflammation, the IFN-inducible gene family 1-8U was overexpressed. However, the consequences of increased IFN-*γ* expression in UC and its contribution to carcinogenesis remain unclear [31].

Other molecules induced by IFN-*γ* have been also observed in IBD patients, such as IL-18 and IL-18 binding protein (IL-18BP), which have been furthermore associated with inflammation and cancer [32].

Interleukin 8 (IL-8), a member of the neutrophil-specific CXC subfamily of chemokines with the ELR (Glu-Leu-Arg) motif, acts as a chemoattractant to neutrophils during acute inflammatory response [206]. Increased levels of this chemokine have been reported in IBD patients [207], correlating histologically with areas of active inflammation [208], mainly associated with neutrophils and macrophages [209]. Additionally, colon cancer cells also express IL-8 [210]; in sporadic cancer, higher levels of this cytokine were observed in tissue from moderately and poorly differentiated as compared to well-differentiated tumors [211]. In addition, IL-8 levels are directly correlated with metastatic potential in colon cancer cell lines [210]. Overexpression of IL-8 in HCT116 and Caco2 cell lines results in increased proliferation, cell migration, and invasion, while in a tumor xenograft model, IL-8-overexpressing cells formed larger tumors and showed higher microvessel density [41]. This* in vivo* effect of IL-8 on angiogenesis is supported by a study using primary cultures of human intestinal microvascular endothelial cells, which respond to IL-8 through the CXCR2 receptor, eliciting an angiogenic response [212].

These findings illustrate the complex role of cytokines in the various events associated with the development of CAC. Therefore, controlling the inflammatory process early in IBD is important for reducing risk of colorectal cancer.

## 6. Primary Sclerosing Cholangitis- (PSC-) and Liver Fluke-Associated Cholangiocarcinoma (CCA)

CCA is a malignant neoplasm originating from the epithelial cells lining the intra- or extrahepatic biliary ducts. It is the second-most frequent liver cancer worldwide, after hepatocellular carcinoma. Five-year survival is about 10%. In the United States, incidence of CCA in the Hispanic population is 2.8 per 100,000; in Asians, 3.3 per 100,000; and in non-Hispanic Caucasians and African-Americans, 2.1 per 100,000 [213]. However, incidence varies widely, from the highest reported rate of 113 per 100,000 in the Khon Kaen province of Thailand to as low as 0.1 per 100,000 in Australia [214, 215].

There are several factors that increase the risk for CCA, including primary sclerosing cholangitis, parasitic infection, biliary-duct cysts, hepatolithiasis, viral infection, and toxins [23, 216]. Primary sclerosing cholangitis (PSC) is characterized by inflammation and fibrosis of biliary ducts leading to biliary tract stricture. The cumulative lifetime incidence of CCA in PSC is around 20% [217]. More than 50% of patients with PSC develop CCA simultaneously or within 1 year of diagnosis [218]. The incidence of CCA after PSC diagnosis has been reported in several studies at around 0.5–1.5% per year [217–219]. CCA must be suspected in any new PSC patient presenting with jaundice, suggesting chronic inflammation of the bile duct.

*Opisthorchis viverrini* (*O. viverrini*) and* Clonorchis sinensis* (*C. sinensis*) have been classified by the International Agency for Research on Cancer (IARC) as Group I (carcinogenic in humans) [220] and as the most common risk factors for CCA, especially in East and Southeast Asia [221, 222]. The high incidence of* O. viverrini *infection, which is due to the custom of eating raw fish containing the infectious stage of the parasites, was found to be correlated with the high prevalence of CCA in the northeastern part of Thailand [221]. PSC, hepatolithiasis, and choledochal cysts are the risk factors for CCA in areas where liver fluke is not endemic in Thailand [215]. In addition, biliary ascariasis caused by* Ascaris lumbricoides* infection in China, India, and some areas of South America has also been reported in association with CCA development [223, 224].

Infection with hepatitis viruses can generate hepatocellular carcinomas, especially hepatitis B, in which more than 80% of cases develop cancer [225]. It is becoming more accepted that both hepatitis B and hepatitis C viruses may be associated with biliary inflammation and can cause CCA. Approximately 13.8% and 1.9% of CCA patients have positive findings for hepatitis B and hepatitis C, respectively [226].

Other etiologies that may or may not cause bile duct obstruction but result in the chronic inflammation of biliary epithelial cells are proposed CCA risk factors, including gallstone formation, choledochoenteric anastomosis, and chemical and radiation exposure [23].

CCA, like many other cancers in that its carcinogenesis is a multistep process, requires interaction between mutated biliary epithelial cells and environmental factors. Many hallmarks of cancer have been proposed, and the list has been continually updated over the years [7]. The genes involved in controlling these properties have been found to be mutated in cancer patients. In CCA, several protooncogenes including K-ras [227–229], c-erbB-2, and c-Met [230]; tumor suppressor genes, that is, p53; and antiapoptotic genes such as Bcl-2, Bcl-X(L), and Mcl-1 [231] are mutated. In PSC-mediated CCA, the mutation was detected in the promoter, leading to the overexpression of p16INK4a and p14ARF cell cycle regulators [232].

During the genesis of CCA, both PSC and parasitic infections cause cholestasis and chronic inflammation of the bile duct, which can induce the epithelial cells to produce a variety of cytokines including IL-6, IL-8, TGF-*β*, TNF-*α*, platelet-derived growth factor (PDGF), and epidermal growth factor (EGF) (Table 2) [23]. The release of IL-6, TGF-*β*, TNF-*α*, and PDGFA is essential for bile duct epithelial cell proliferation. The production of PDGFA and the overexpression of its receptors during cholangiocarcinogenesis in* O. viverrini*-infected hamsters indicate the potential of these molecules to downregulate many antiproliferative factors and promote the angiogenesis pathway [233]. In addition, PDGFA expression in CCA tissue and serum is correlated with patient survival time and has been proposed as a marker of poor prognosis [234].

TNF-*α* and IFN-*γ*, which are cytokines released during chronic inflammation, can cause alteration of biliary barrier function [24], whereas proinflammatory cytokines alter cholangiocyte choleretic activity [42, 43]. When cholangiocytes are exposed to these cytokines, they respond by secreting other molecules such as IL-8, MCP-1, and CCL-28 that can promote leukocyte adhesion and retention at the site of inflammation, leading to more damage of biliary cells. The injured cholangiocytes can release insulin-like growth factor-1 (IGF-1) and VEGF to stimulate CCA cell growth and angiogenesis, respectively [235–238].

TNF-*α* can activate increased expression of AID (activation-induced cytidine deaminase, a member of the DNA/RNA-editing enzyme family) in CCA-derived cells, but not in PSC-derived epithelial cells [25]. AID results in the generation of somatic mutations of many tumor-related genes, including p53, c-Myc, and CDKN2A (or INK4A/p16) promoter sequences. This finding suggests a connection between chronic inflammation and tumorigenesis via the mutagenic activity of AID [25]. In addition, NF-*κ*B activation in cells by chronic inflammation-derived cytokines might lead to the activation of active transcription factors translocating into the nucleus and regulating the expression of IL-6, TNF-*α*, and several growth factors which can change the microenvironment for tumor promotion [36]. Moreover, the release of nitric oxide with the formation of 3-nitrotyrosine and other reactive oxidants can inhibit the DNA-repair process, which allows for oxidative DNA damage to cells and thus promotes tumor formation [239].

Cholangiocytes and CCA cells do not act alone but are surrounded by several types of cells, generally known as microenvironmental cells. Fibroblasts are the main microenvironmental cells, and their function in stimulating the acquired hallmark capabilities of cancer cells is well-known [240]. Activated CCA-associated fibroblast phenotypes were found to show increased expression of *α*-SMA [241]. Interestingly, these fibroblasts were isolated from CCA tissues obtained from patients and mapped for the specific gene expression pattern resulting in the expression of several cancer-promoting proteins [242]. Researchers have since identified several substances that can be produced by CCA-associated fibroblasts, including periostin, hepatocyte growth factor (HGF), tenascin-C, and CXCL-12 [243, 244]. Interestingly, these soluble factors are involved in several tumorigenic properties leading to the progression and metastasis of the cancer. These findings suggest that fibroblasts, their secreting products, and the activated pathways in the cancer cells could be promising targets for attenuation of disease progression [243, 245].

Many immune cells are known to surround cancer cells, with detrimental or beneficial effects on cancer progression, depending on the profile of substances secreted into the tumor microenvironment. The substances secreted from CCA cells were studied* in vitro* with human macrophages, and the results exhibited M2 polarization of macrophages as well as overproduction of cytokines and other bioactive molecules, including IL-10, VEGF-A, TGF-*β*, and matrix metalloproteinase- (MMP-) 2 [45]. In intrahepatic CCA, the tumor-infiltrating lymphocytes IL-17^+^ and FOXP3^+^, CD66b^+^ neutrophils, and microvessels were predominantly found in the intratumor area, whereas CD8^+^ lymphocytes were most abundant in the tumor invasive front [48]. Although IL-17 levels have never been reported for CCA, this study suggested for the first time that intratumor IL-17^+^ lymphocytes and neutrophils could be used as a marker of poor prognosis in CCA.

TGF-*β* was studied with CCA cell lines, and the results demonstrated the potential of TGF-*β* to induce EMT-mediated cancer progression via the Snail transcription factor, leading to increasing levels of vimentin, S100A4, collagen type 1, and MMP-2 production [40]. EMT level is closely associated with aggressiveness of the disease and could be proposed as a marker of poor prognosis. Moreover, TNF-*α* has been recently reported to have the ability to induce EMT of CCA cells [26].

In conclusion, the chronic inflammation-driven cytokines released from biliary cells, fibroblasts, or immune cells into the microenvironment of the bile duct epithelium may facilitate cell immortalization, evasion of apoptosis, and autonomous proliferation in untransformed cells, leading to the development of CCA [23]. In addition, cytokines may help activate invasion, metastasis, and EMT-mediated CCA progression.

## 7. Conclusion

The tumor microenvironment formed by stromal cells, infiltrating immune cells, and tumor cells contains factors that can promote carcinogenesis. Ample evidence supports the involvement of cytokines in events leading to the initiation, promotion, invasion, and metastasis of cancer (Figure 1). In a chronic inflammatory process, cytokines such as TNF-*α* and IL-6 induce the generation of free radicals that can damage DNA, potentially causing mutations that lead to tumor initiation. Tumor growth is also favored by proinflammatory cytokines that stimulate cell proliferation and reduce apoptosis, while anti-inflammatory cytokines, such as IL-10 and TGF-*β*, contribute to tumor immune evasion. The invasive properties of tumors are related to the activation of the epithelial-mesenchymal transition program triggered by TGF-*β* and enhanced by proinflammatory cytokines, such as TNF-*α* and IL-6. Proinflammatory cytokines also play an important role in angiogenesis and metastasis. In the latter, chemokines such as IL-8 have an important role in cell migration to other tissues.

Although we observed that many cytokines contribute to carcinogenesis, their pro- or antitumoral roles depend on the balance of these different inflammatory mediators and the stage of tumor development. For this reason, studying the role of these mediators in different tumors or stages of development is essential for designing new personalized treatments using these potential therapeutic targets.

In this line, the potential role of cytokines has been reported, as a diagnostic marker for cancer. The determination of the serum levels of cytokines, such as IL-6 or IL-10, might be associated with a tumorigenic process or poor prognosis [69, 105]. However, further prospective studies are needed to determine trusted cut-off values of circulating cytokine to establish a direct relationship with cancer.

In the field of therapy, several clinical trials have been implemented in order to evaluate inhibitors of cytokines receptors or neutralizing antibodies that prevent the sustained exposure to these inflammatory mediators that promote tumor progression [80, 103]. On the other hand, from the findings of Coley [56], who associates an infectious process with the control of tumor progression, arises the idea to cause an acute inflammation to activate antitumor response mechanisms [58].

While progress has been made in the understanding of the mechanisms of these cytokines in the tumorigenic process, establishing a relationship between cytokines expression and disease progression, survival, and response to therapy remains a major challenge.

## Figures and Tables

**Table 1:** Cancer associated with chronic inflammatory disorders.

Cancer	Associated inflammatory stimuli	Reference
Colorectal cancer/colitis-associated cancer	Inflammatory bowel diseases (ulcerative colitis and Crohn's diseases)	[8]
Cholangiocarcinoma	Liver fluke and primary sclerosing cholangitis	[9]
Gastric cancer	Chronic gastritis (*H. Pylori*)	[10]
Lung cancer	Inflammation caused by asbestos, infections, smoking, and silica	[11]
Prostate cancer	*E. coli* infection of prostate	[12]
Hepatocellular carcinoma	Infection with hepatitis virus B and hepatitis virus C	[13]
Melanoma	UV irradiation-associated skin inflammation	[14]
Endometrial carcinoma	Endometriosis	[15]
Gall bladder carcinoma	Gall bladder stone-associated chronic cholecystitis	[16, 17]
Esophageal cancer	Barrett's esophagitis	[18]

**Table 2:** Significance and role of cytokines in tumorigenesis.

Cytokines	Colitis-associated cancer (references)	CCA (references)
TNF-*α*	Tumor-promoting role in various stages of carcinogenesis. Related to RONS generation in IBD patients, promoting oxidative stress-mediated DNA damage. Stimulates TGF-*β*-induced EMT. Induces secretion of VEGF by human fibroblasts, promoting angiogenesis. Induces NF-κB signaling, a decisive pathway in driving metastasis in a model of CAC [19–22].	Essential for bile duct epithelial cell proliferation. Impairs epithelial barrier function. Disrupts cholangiocyte tight-junction and influences the aggravation of bile duct cholestasis. Induces a DNA/RNA-editing enzyme (AID) in CCA cells, resulting in somatic mutation of several tumor-related genes and leading to cholangiogenesis. EMT induction in CCA cells *in vitro* [23–26].

IFN-*γ*	Increases in IFN-*γ* ^+^ cells have been observed in IBD patients. Deficient mice did not develop DSS-induced colitis. Increases paracellular permeability in early IBD pathogenesis. Deficient mice developed higher numbers of tumors, suggesting an antitumor immune response of IFN-*γ*. In patients with UC-associated cancer and a group of UC patients with chronic severe inflammation, the IFN-inducible gene family 1-8U was overexpressed. Induces IL-18 and IL-18 binding protein (IL-18BP) in IBD, which have been also associated with inflammation and cancer [27–32].	Reduces transepithelial electrical resistance. Alters cholangiocyte tight-junction, leading to aggravation of bile duct cholestasis [24].

IL-6	Induces oxidative stress. A critical tumor promoter during early CAC tumorigenesis. TAM-derived IL-6 contributes to CAC in animal models. CRC patients present with high levels of IL-6 and VEGF [19, 33–35].	Cholangiocyte and CCA cells can be activated by proinflammatory cytokines through the NF-*κ*B-dependent pathway, leading to overproduction of bile duct epithelium growth factor, thus promoting cancer initiation and progression [36, 37].

TGF-*β*	Induces CAC progression, promoting EMT. In later stages of carcinogenesis, it promotes tumor growth by creating an immunotolerant tumor environment [38, 39].	Promotes proliferation of bile duct epithelial cells and induces EMT-mediated tumor aggressiveness [23, 40].

IL-8	Colon cancer cell lines overexpressing IL-8 show enhanced proliferation, migration, and angiogenesis. IL-8 induced by TNF-*α* accelerates EMT [21, 41].	Secreted by cholangiocytes in response to proinflammatory cytokines and together with MCP-1 and CCL-28 promotes leukocyte adhesion and retention in injured biliary epithelial cells. Injured cholangiocytes then release IGF-1 and VEGF, which can stimulate CCA cell growth [42, 43].

IL-10	IL-10^−/−^ mice develop colitis and colorectal cancer, similar to IBD-associated cancer in humans [44].	CCA can activate macrophage polarization into M2 phenotype through the STAT-3 pathway, leading to IL-10, VEGF-A, TGF-*β*, and MMP-2 production [45].

IL-17	Overexpressed in tumors from CAC patients and is associated with angiogenesis and poor prognosis markers. Secreted in tumors by macrophages/monocytes CD68+; Th17 and Treg FOXP3^+^IL17^+^ cells [46, 47].	Tumor-infiltrating lymphocytes IL-17^+^ are found in CCA intratumoral areas and correlate with lymph node metastasis, intrahepatic metastasis, and advanced stages [48].

IL-21	Enhanced in mucosa of IBD patients and in the CAC mouse model. Blockade of IL-21 signaling reduces tumor development and mucosal microenvironment inflammation [49].	No available references for this cytokine in CCA.
